# Beyond LDL-C: Biomarker-Informed Risk Assessment and Management of Residual Cardiovascular Risk in the Era of Precision Prevention

**DOI:** 10.3390/diagnostics16213480

**Published:** 2026-10-24

**Authors:** Shefa Arya Nezhad, Armin Nouri, Laura Moreines, Sridhar Mangalesh, Parvaneh Rastgou, Mohnish Maheshwari, Raiza Rossi, Abdulla Damluji, Michael G. Nanna

**Affiliations:** 1 Section of Cardiovascular Medicine, Yale School of Medicine, 333 Cedar Street, New Haven, CT 06510, USA; aryanezhad.shefa@gmail.com (S.A.);; 2 Jacobi Medical Center, Albert Einstein College of Medicine, 1400 Pelham Parkway South, Bronx, NY 10461, USA; 3 Farmington High School, 14 Monteith Drive, Farmington, CT 06032, USA; 4 The Cardiovascular Center on Aging, Department of Cardiovascular Medicine, Cleveland Clinic, Cleveland, OH 44195, USA

**Keywords:** residual cardiovascular risk, LDL cholesterol, apolipoprotein B, lipoprotein(a), high-sensitivity C-reactive protein, precision prevention, biomarkers

## Abstract

Despite substantial reductions in atherosclerotic cardiovascular disease (ASCVD) events with intensive low-density lipoprotein cholesterol (LDL-C) lowering, considerable residual cardiovascular risk persists among individuals achieving guideline-recommended LDL-C targets. This residual risk reflects biological pathways not addressed by LDL-C lowering and remains a central challenge in preventive cardiology. This narrative review examines three overlapping domains: residual lipid risk related to triglyceride-rich lipoprotein remnants and excess apolipoprotein B-containing particles; genetically determined lipoprotein(a) [Lp(a)]-mediated risk; and residual inflammatory risk involving the NLRP3 inflammasome and downstream interleukin-1β and interleukin-6 signaling. Complementary biomarkers, including apolipoprotein B, non-HDL cholesterol, remnant cholesterol, Lp(a), and high-sensitivity C-reactive protein, may help characterize residual-risk phenotypes beyond LDL-C alone, although their incremental value and influence on management vary with the strength of prognostic and causal evidence and the availability of effective therapies. Icosapent ethyl and low-dose colchicine have demonstrated cardiovascular benefit in selected high-risk populations, whereas the cardiovascular benefits of therapies targeting apoC-III, ANGPTL3, and Lp(a) remain under evaluation. Recent phase 3 findings have further underscored uncertainty regarding the cardiovascular benefit of interleukin-6 inhibition. Integrating complementary biomarkers with therapies supported by randomized outcomes evidence may enable a more individualized approach to residual cardiovascular risk reduction.

## 1. Introduction

Cardiovascular disease, largely driven by atherosclerotic cardiovascular disease (ASCVD), remains a leading cause of morbidity and mortality worldwide, and lowering low-density lipoprotein cholesterol (LDL-C) has been a cornerstone of ASCVD prevention for more than three decades [1,2,3]. The causal relationship between LDL-C and atherosclerosis is supported by convergent epidemiologic, genetic, and randomized evidence; meta-analyses of randomized trials indicate that each 1 mmol/L (approximately 39 mg/dL) reduction in LDL-C is associated with an approximately 22% relative reduction in major vascular events [3,4]. These findings have driven progressive intensification of lipid-lowering therapy, including combination regimens with ezetimibe, proprotein convertase subtilisin/kexin type 9 (PCSK9) inhibitors, bempedoic acid, and inclisiran [2]. Contemporary guidelines therefore emphasize intensive LDL-C lowering, with long-term follow-up studies supporting the safety of achieving levels well below historically accepted targets [2,5].

Despite these advances, a substantial burden of cardiovascular events persists among patients who achieve guideline-recommended LDL-C levels [6,7]. Trials of ezetimibe and PCSK9 inhibition have shown that progressively lower LDL-C reduces cardiovascular events but does not eliminate recurrent risk, implicating additional pathways not addressed by LDL-C lowering [6,7,8,9].

Among the multiple contributors to residual cardiovascular risk [9], this review focuses on three overlapping biological domains for which complementary biomarkers and mechanism-specific therapeutic strategies are particularly relevant: (1) residual lipid risk reflecting persistent exposure to atherogenic lipoproteins not fully captured or addressed by LDL-C lowering [9,10]; (2) lipoprotein(a) (Lp(a))-mediated risk representing a genetically determined and largely treatment-resistant source of cardiovascular risk [11,12]; and (3) residual inflammatory risk involving persistent activation of pathways that include the NLR family pyrin domain containing 3 (NLRP3) inflammasome and interleukin-1β (IL-1β)–interleukin-6 (IL-6)–C-reactive protein (CRP) signaling axis [13,14]. Rather than attempting to catalog all determinants of residual clinical risk, including hypertension, diabetes, obesity, smoking, and chronic kidney disease, this review focuses on selected biological pathways that persist despite LDL-C optimization and their relevance to biomarker-based risk characterization and therapeutic development [2,9,15]. Recognition of these distinct but overlapping pathways has spurred interest in complementary biomarkers that may identify the predominant drivers of persistent risk [9].

This review offers a clinically oriented framework for residual cardiovascular risk in the era of intensive LDL-C lowering. It distinguishes biomarkers associated with residual risk, biological pathways supported by causal evidence, and therapeutic strategies validated by randomized cardiovascular outcomes trials. We examine established and emerging biomarkers, evaluate their clinical applicability after LDL-C goal achievement, and summarize current and emerging therapies according to the strength of evidence supporting their use.

## 2. Methods

This narrative review was informed by a targeted search of PubMed/MEDLINE, major clinical practice guidelines, randomized clinical trials, meta-analyses, consensus statements, and high-impact peer-reviewed publications addressing residual cardiovascular risk, associated biomarkers, and emerging therapeutic strategies. Search concepts included residual cardiovascular risk, residual lipid risk, remnant cholesterol, apolipoprotein B (apoB), lipoprotein(a) [Lp(a)], high-sensitivity C-reactive protein (hsCRP), inflammation, triglyceride-rich lipoproteins, icosapent ethyl (IPE), colchicine, apolipoprotein C-III (apoC-III), angiopoietin-like protein 3 (ANGPTL3), interleukin-6 inhibition, and emerging cardiovascular biomarkers. Priority was given to publications most relevant to the clinical questions addressed in this review, emphasizing clinical practice guidelines, major randomized trials, consensus statements, and landmark studies that established key biological and therapeutic concepts. The literature was updated through August 2026.

During the preparation of this manuscript, the authors used ChatGPT (GPT-5.6, OpenAI) to identify grammatical errors and improve language clarity. All ChatGPT-assisted suggestions were reviewed and edited by the authors, who take full responsibility for the content of the manuscript.

## 3. Defining Residual Cardiovascular Risk

Although no universally accepted definition exists, residual cardiovascular risk generally refers to the ongoing risk of incident or recurrent ASCVD events despite appropriate management of established cardiovascular risk factors and implementation of guideline-directed preventive therapies [16]. It should be distinguished from risk attributable to incomplete implementation of preventive therapy or failure to achieve recommended LDL-C goals [2,16]. Some patients remain at high risk because LDL-C has not been sufficiently reduced, whereas others continue to experience cardiovascular events despite achievement of recommended LDL-C levels and control of conventional risk factors [17].

Persistent risk despite intensive LDL-C lowering is evident in secondary-prevention trials, including the Improved Reduction of Outcomes: Vytorin Efficacy International Trial (IMPROVE-IT), Further Cardiovascular Outcomes Research with PCSK9 Inhibition in Subjects with Elevated Risk (FOURIER), and Evaluation of Cardiovascular Outcomes After an Acute Coronary Syndrome During Treatment with Alirocumab (ODYSSEY OUTCOMES) trials [6,7,8]. Residual risk is a heterogeneous construct arising from multiple, overlapping biological pathways [16,17]. However, the clinical relevance of biomarkers such as apoB, remnant cholesterol, Lp(a), and hsCRP depends on whether they provide actionable information and whether the corresponding pathway has evidence supporting therapeutic intervention [10,11,14,17]. For this review, residual cardiovascular risk is considered across three overlapping biological domains: residual lipid risk, Lp(a)-mediated risk, and residual inflammatory risk. This framework highlights selected biological contributors that persist despite LDL-C optimization and complements the traditional phenotypic and behavioral risk factors emphasized by prevention guidelines, rather than providing an exhaustive classification of residual cardiovascular risk.

## 4. Biological Mechanisms of Residual Cardiovascular Risk

**Figure 1:**
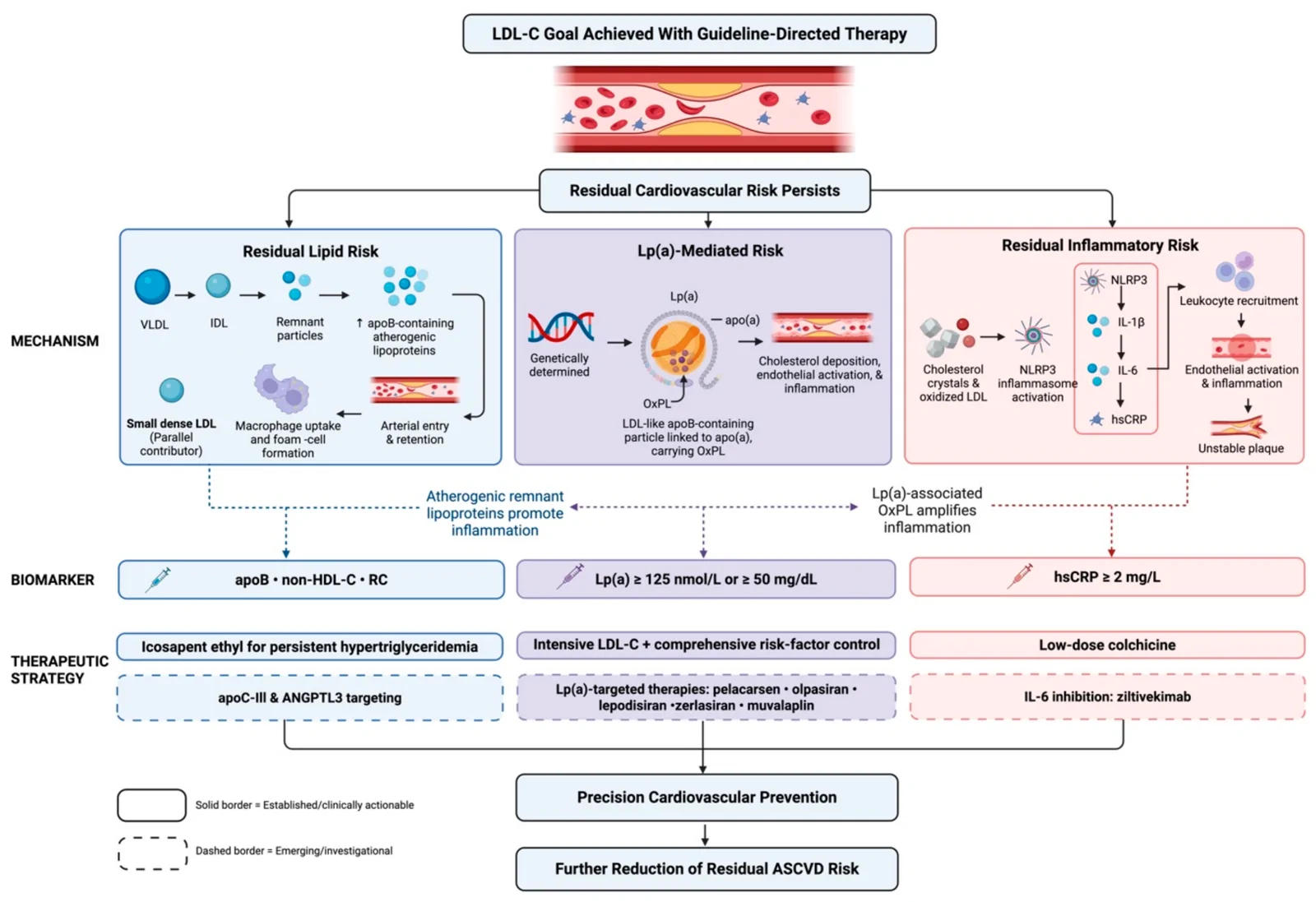
Central Illustration. Biomarker-informed precision cardiovascular prevention for residual cardiovascular risk despite achievement of LDL-C goals. **Abbreviations:** LDL-C, low-density lipoprotein cholesterol; VLDL, very-low-density lipoprotein; IDL, intermediate-density lipoprotein; apoB, apolipoprotein B; non-HDL-C, non-high-density lipoprotein cholesterol; Lp(a), lipoprotein(a); apo(a), apolipoprotein(a); OxPL, oxidized phospholipids; NLRP3, NLR family pyrin domain containing 3; IL-1β, interleukin-1 beta; IL-6, interleukin-6; hsCRP, high-sensitivity C-reactive protein; apoC-III, apolipoprotein C-III; ANGPTL3, angiopoietin-like protein 3; ASCVD, atherosclerotic cardiovascular disease; RC, remnant cholesterol.

The following sections summarize the clinically relevant biological basis of the three residual-risk domains introduced above. Their relationships with representative biomarkers and therapeutic strategies are presented in the Central Illustration (Figure 1).

### 4.1. Residual Lipid Risk

Residual lipid risk reflects persistent exposure to atherogenic lipoproteins despite achievement of recommended LDL-C levels [10,18]. As illustrated in Figure 1, triglyceride-rich lipoproteins (TRLs), including very-low-density lipoproteins, intermediate-density lipoproteins, and chylomicron remnants, undergo lipolysis to generate cholesterol-enriched remnant particles that may enter the arterial wall, promote macrophage lipid accumulation, and contribute to plaque progression [18,19,20]. Genetic and epidemiologic evidence supports a causal contribution of apoB-containing triglyceride-rich remnant particles to ASCVD [21,22,23]. However, randomized cardiovascular outcomes trials of therapies targeting triglyceride and remnant metabolism have produced heterogeneous results [24,25].

Qualitative differences in LDL particles may also contribute to residual lipid risk [10]. Small dense LDL particles have properties that enhance atherogenicity, including greater arterial-wall penetration and susceptibility to oxidative modification [19,26]. Elevated small dense LDL is consistently associated with ASCVD in observational studies and is common in insulin resistance and metabolic syndrome; however, its independent causal contribution and clinical utility as a routine biomarker or therapeutic target remain uncertain [19,26].

Residual lipid risk may also arise from discordance between LDL-C concentration and the number of circulating atherogenic particles [27,28]. Because each apoB-containing lipoprotein carries a single apoB molecule, apoB reflects total atherogenic particle burden and explains discordance between cholesterol concentration and particle number [27].

### 4.2. Lipoprotein(a)-Mediated Residual Risk

Lp(a) is a genetically determined contributor to residual cardiovascular risk that consists of an LDL-like apoB-containing particle covalently linked to apolipoprotein(a) (apo(a)) [11]. Its circulating concentration is predominantly determined by variation in the LPA gene, varies substantially among individuals, and is minimally influenced by lifestyle modification or conventional lipid-lowering therapy [11,29]. Epidemiologic, Mendelian randomization, and human genetic studies support a causal relationship between elevated Lp(a) and ASCVD [11,12,30]. Elevated Lp(a) also remains associated with cardiovascular risk among patients receiving intensive LDL-C-lowering therapy [31].

The cardiovascular effects of Lp(a) appear to extend beyond the cholesterol carried by its LDL-like component [11,32]. Lp(a) carries oxidized phospholipids that have been implicated in endothelial activation, inflammatory signaling, monocyte recruitment, and foam-cell formation [32,33]. These mechanisms may contribute to both atherosclerosis and calcific aortic valve disease [11,34]. Structural homology between apo(a) and plasminogen has also suggested potential effects on fibrinolysis, although the contribution of this proposed prothrombotic mechanism to clinical cardiovascular risk remains uncertain [11,35].

### 4.3. Residual Inflammatory Risk

Persistent vascular inflammation may contribute to residual cardiovascular risk through activation of inflammatory pathways involved in atherosclerosis [36,37]. Experimental and translational studies implicate activation of the NLRP3 inflammasome and downstream IL-1β–IL-6–CRP signaling in atherogenesis and progression of vascular inflammation [13,37]. Cholesterol crystals, oxidized lipoproteins, and cellular debris may activate this pathway, while diabetes and impaired cholesterol efflux may provide additional pro-inflammatory stimuli [13,38]. Clonal hematopoiesis represents another inflammatory stimulus linked to accelerated atherosclerosis [39,40].

Inflammation also interacts closely with thrombosis through the related concepts of immunothrombosis and thromboinflammation [41]. Immunothrombosis refers to the coordinated activation of innate immune pathways, platelets, and coagulation as part of a physiological host-defense response to infection or tissue injury [41,42]. When excessive or dysregulated, these interactions may manifest as thromboinflammation, characterized by reciprocal activation of leukocytes, platelets, endothelial cells, complement, and the coagulation system, thereby promoting pathological thrombosis and vascular injury [43]. In ASCVD, these processes may contribute to the thrombotic complications of plaque disruption, providing an additional mechanistic link between vascular inflammation and atherothrombotic events [41,44].

Randomized cardiovascular outcomes trials of selected anti-inflammatory interventions support inflammation as a potentially modifiable contributor to ASCVD, although cardiovascular benefit has not been consistent across anti-inflammatory strategies or clinical settings [14,45,46]. Nevertheless, lipid-mediated and inflammatory pathways remain closely interconnected: atherogenic lipoproteins may activate innate immune signaling, while inflammatory mediators may promote lipid retention and plaque progression [38,47]. Clonal hematopoiesis may further amplify inflammasome and IL-6 signaling, and the oxidized phospholipid content of Lp(a) may provide an additional link between lipid-mediated and inflammatory risk [11,32,33].

## 5. Biomarkers for Identifying Residual Risk

Because no single biomarker captures all residual-risk pathways, complementary measurements may provide more comprehensive risk characterization [47,48]. Although LDL-C remains the primary therapeutic target in contemporary lipid management, complementary biomarkers may provide additional information when residual atherogenic burden is not fully captured by conventional lipid measures; however, their clinical utility varies according to the strength of evidence supporting their use in risk assessment and management [49]. The clinical characteristics, strengths, limitations, and supporting evidence for these biomarkers are summarized in Table 1.

### 5.1. Apolipoprotein B and Non-HDL Cholesterol

Among biomarkers used to assess residual lipid risk, apoB is one of the most clinically validated measures of atherogenic lipoprotein burden [19,27]. Elevated apoB is strongly associated with ASCVD risk and reflects the burden of apoB-containing particles, which are causal in atherosclerosis [19,27]. ApoB may provide additional information beyond LDL-C, particularly when cholesterol content and particle number are discordant [27,28]. The 2026 American College of Cardiology/American Heart Association (ACC/AHA) multisociety dyslipidemia guideline considers apoB measurement reasonable in adults receiving lipid-lowering therapy, particularly those with ASCVD, cardiovascular-kidney-metabolic syndrome, type 2 diabetes, and/or elevated triglycerides, to guide decisions regarding further therapeutic intensification once LDL-C and/or non-HDL-C goals have been achieved [2]. However, although apoB reflects atherogenic particle burden, randomized trials have not directly established that an apoB-guided treatment strategy improves cardiovascular outcomes compared with treatment strategies based on conventional lipid measures [27].

Non-high-density lipoprotein cholesterol (non-HDL-C) reflects the cholesterol content of all apoB-containing lipoproteins and provides a practical measure of total atherogenic cholesterol burden [27,50]. It is particularly useful in patients with elevated triglycerides, in whom LDL-C may incompletely capture triglyceride-rich atherogenic cholesterol burden [2]. The 2026 ACC/AHA multisociety guideline recognizes non-HDL-C as an important measure of atherogenic cholesterol burden and incorporates non-HDL-C treatment goals alongside LDL-C targets in selected clinical settings [2]. Because it requires no testing beyond a routine lipid panel, non-HDL-C remains an accessible measure of residual atherogenic cholesterol burden [66,67,68].

### 5.2. Remnant Cholesterol

Remnant cholesterol (RC) represents the cholesterol carried by triglyceride-rich lipoproteins, including very-low-density lipoproteins, intermediate-density lipoproteins, and chylomicron remnants, and is commonly estimated as non-HDL-C minus LDL-C [57,69,70]. Elevated RC is particularly common in individuals with insulin resistance, type 2 diabetes, obesity, metabolic syndrome, and hypertriglyceridemia and is associated with increased risk of ASCVD events, including myocardial infarction and major adverse cardiovascular events (MACE), even after accounting for LDL-C levels [21,57,71,72,73]. RC measurement may therefore complement assessment of triglyceride-rich lipoprotein burden, although its clinical role is less established than that of apoB or non-HDL-C [2,74].

Although genetic and epidemiologic evidence supports a causal role for remnant lipoproteins in ASCVD, RC currently serves primarily as a biomarker of this pathway rather than an established therapeutic target [18,21,57]. Its routine use is limited by indirect estimation, the absence of universally accepted diagnostic thresholds and treatment targets, and the lack of randomized evidence demonstrating that treatment selected or titrated according to RC concentration reduces cardiovascular events [18,74]. Ongoing clinical development of therapies targeting apoC-III and ANGPTL3 will further define their role in remnant-lipoprotein disorders, although whether these approaches reduce ASCVD events remains to be established [18,74].

### 5.3. Lipoprotein(a)

Lp(a) is a genetically determined apoB-containing lipoprotein that represents a causal pathway in ASCVD and serves as an established biomarker of residual cardiovascular risk [52]. Its circulating concentration is predominantly determined by variation in the LPA gene, remains relatively stable throughout life, and is minimally influenced by lifestyle modification or conventional lipid-lowering therapies [2,52]. Epidemiologic, Mendelian randomization, and genetic studies support a causal relationship between elevated Lp(a) concentrations and ASCVD risk [52].

Lp(a) measurement provides information beyond conventional lipid measures by identifying inherited cardiovascular risk that is not captured by LDL-C or other routinely available lipid parameters [2]. Current major lipid guidelines recommend measuring Lp(a) at least once during adulthood, with particular clinical relevance in individuals with premature ASCVD, recurrent cardiovascular events despite LDL-C lowering, familial hypercholesterolemia, premature calcific aortic valve disease, or a strong family history of premature ASCVD [2]. Identification of elevated Lp(a) should prompt optimization of LDL-C lowering and other modifiable cardiovascular risk factors; however, current management is not specifically directed at lowering Lp(a) because no Lp(a)-lowering therapy has yet demonstrated cardiovascular outcomes benefit [2].

Despite strong causal evidence, Lp(a) currently functions as a risk-stratification biomarker rather than an outcomes-validated therapeutic target [2,52]. Randomized trials have not established that Lp(a)-guided treatment strategies improve cardiovascular outcomes compared with conventional risk-factor management [2,75]. PCSK9 monoclonal antibodies modestly reduce Lp(a), whereas RNA-targeted therapies, including antisense oligonucleotides and small interfering RNAs (siRNAs), produce substantially greater reductions and are being evaluated in cardiovascular outcomes trials (Section 6.3) [31,52,75,76,77,78,79,80,81].

### 5.4. High-Sensitivity C-Reactive Protein (Residual Inflammatory Risk)

High-sensitivity C-reactive protein (hsCRP), produced primarily by the liver in response to interleukin-6 (IL-6) signaling, is a widely studied and clinically available biomarker of residual inflammatory risk [53,56]. In clinically stable individuals, hsCRP shows long-term biological stability and provides prognostic information beyond traditional risk factors, including LDL-C [56,82,83].

Elevated hsCRP is associated with increased cardiovascular risk independently of achieved LDL-C concentrations and identifies individuals with a higher-risk inflammatory profile despite contemporary preventive therapy [2,84]. However, hsCRP should be interpreted primarily as a marker of inflammatory risk rather than evidence of a causal inflammatory pathway that can be directly targeted [85]. In primary prevention, current guidelines recognize elevated hsCRP as a risk-enhancing factor that may inform statin decision-making in selected individuals, whereas its role in guiding anti-inflammatory treatment in secondary prevention remains incompletely established [53].

In the Canakinumab Anti-inflammatory Thrombosis Outcomes Study (CANTOS), greater on-treatment hsCRP reduction was associated with greater cardiovascular benefit, supporting hsCRP as a potential response biomarker rather than a therapeutic target itself [86]. Although hsCRP may characterize residual inflammatory risk, no randomized trial has established an hsCRP-guided treatment strategy that improves cardiovascular outcomes [53].

### 5.5. Emerging Biomarkers

Although current clinical practice relies primarily on established biomarkers such as apoB, non-HDL-C, remnant cholesterol, Lp(a), and hsCRP, several emerging biomarkers and multi-omic technologies may further refine residual cardiovascular risk stratification [54,87]. These approaches capture complementary biological processes beyond conventional lipid and inflammatory pathways and may improve identification of individuals at increased residual cardiovascular risk, rather than establish causal mechanisms or validated therapeutic targets [54,87]. At present, however, these approaches remain largely investigational [54,87].

*GlycA:* GlycA is a nuclear magnetic resonance (NMR)-derived composite biomarker reflecting N-acetyl glycan moieties on circulating acute-phase proteins and provides an integrated measure of systemic inflammation [58,88]. Prospective cohort studies have shown that elevated GlycA is associated with incident ASCVD, coronary artery calcium progression, and MACE [88,89,90]. GlycA and hsCRP provide independent and additive prognostic information, suggesting that they capture partly distinct aspects of inflammatory risk [91]. Whether GlycA identifies a causal inflammatory pathway or can be used to guide targeted therapy remains uncertain [58]. Accordingly, current evidence does not support routine clinical measurement outside research settings [58].

*Proteomics:* Proteomic profiling enables the simultaneous measurement of hundreds to thousands of circulating proteins, providing insights into the biological pathways linking inflammation, metabolism, and cardiovascular disease [87]. Representative proteins identified in cardiovascular proteomic studies include growth differentiation factor-15 (GDF-15), N-terminal pro-B-type natriuretic peptide (NT-proBNP), cardiac troponins, and interleukin-6 [92,93]. Large population-based studies have demonstrated that proteomic risk scores improve cardiovascular risk discrimination beyond traditional clinical models and may further improve individualized cardiovascular risk prediction when integrated with clinical and genetic risk information [94,95]. At present, these protein signatures primarily serve as biomarkers for risk stratification, and measurement currently does not alter routine clinical management [54,87].

*Metabolomics:* Metabolomic profiling identifies circulating molecular signatures reflecting lipid, amino-acid, energy, and inflammatory pathways associated with cardiovascular disease [60]. Specific examples associated with cardiovascular risk include branched-chain amino acids, acylcarnitines, trimethylamine N-oxide (TMAO), and ceramide species [64,65]. Integration of metabolomic biomarkers with conventional risk models can provide incremental predictive information, although the magnitude of improvement varies across populations, assays, and modeling approaches [54,96]. The causal significance and therapeutic implications of most metabolomic signatures remain uncertain, and routine metabolomic profiling has not yet been established for cardiovascular treatment decisions [54,97].

*Polygenic Risk Scores (PRS):* These scores estimate inherited susceptibility to ASCVD by integrating the cumulative effects of thousands of common genetic variants [55]. Coronary artery disease polygenic risk scores (CAD-PRS), for example, can identify individuals with elevated inherited susceptibility to ASCVD and may provide additional risk information beyond conventional clinical assessment in selected populations [55,59]. PRS may be particularly valuable in younger individuals, in whom traditional risk factors may underestimate lifetime cardiovascular risk, and may further enhance risk stratification when combined with clinical and multi-omic biomarkers [55,96]. Unlike circulating biomarkers, PRS reflects inherited susceptibility rather than a dynamic residual-risk state [55]. As genomic risk markers, PRS may help identify individuals at increased inherited cardiovascular risk but do not themselves represent modifiable therapeutic targets [55].

*NETosis-related biomarkers:* Neutrophil extracellular traps (NETs) are extracellular networks of chromatin, histones, and neutrophil-derived proteins involved in vascular inflammation and thrombosis [61]. Circulating surrogate markers used to assess NET formation include citrullinated histone H3, myeloperoxidase–DNA complexes, neutrophil elastase–DNA complexes, nucleosomes, and cell-free DNA [62,63]. These markers have been investigated in coronary artery disease and acute myocardial infarction as indicators of thromboinflammatory activity [61,98]. However, assay standardization remains limited, and some markers, particularly cell-free DNA, are not specific to NET formation; therefore, NET-related biomarkers remain investigational [62].

Collectively, these emerging biomarkers and multi-omic technologies have the potential to refine residual cardiovascular risk stratification beyond conventional biomarkers, particularly through integrated precision medicine approaches that incorporate complementary biological dimensions of residual cardiovascular risk [54,87]. Standardization, external validation, affordability, and demonstration of incremental clinical utility remain necessary before these technologies can enter routine cardiovascular practice [54].

### 5.6. Clinical Relevance of Biomarkers

Collectively, these biomarkers provide complementary information about distinct biological pathways contributing to residual cardiovascular risk; however, their clinical interpretation depends on the strength of evidence supporting prognostic value, causal relevance, and therapeutic actionability [54,87]. Among currently available biomarkers, apoB and Lp(a) have the strongest evidence linking their measured concentrations to atherosclerotic risk, with substantial genetic and epidemiologic support for causal relevance [19,27,52]. Remnant cholesterol and hsCRP provide useful risk characterization, although their roles as direct treatment-selection biomarkers remain less well established [18,53,74,86]. Emerging biomarkers, including GlycA, proteomic and metabolomic signatures, PRS, and NETosis-related biomarkers, remain primarily investigational and require further validation before routine clinical use [54,55,62,87,97].

## 6. Therapeutic Strategies Targeting Residual-Risk Pathways

Recognition of the distinct biological mechanisms underlying residual cardiovascular risk has important therapeutic implications [2,53]. After optimization of LDL-C-lowering therapy, treatment may be considered according to the biological pathways contributing to residual risk and the strength of evidence supporting available therapies [2,53].

### 6.1. Targeting Residual Lipid Risk

Among residual lipid pathways, therapeutic relevance should be prioritized according to evidence from randomized cardiovascular outcomes trials rather than biomarker improvement alone [2]. Persistent hypertriglyceridemia and elevated remnant cholesterol characterize a residual lipid phenotype that is particularly common in patients with diabetes, obesity, insulin resistance, and metabolic syndrome [18]. Among currently available therapies, IPE has robust randomized evidence for reducing cardiovascular events among statin-treated patients with persistent hypertriglyceridemia, with evidence derived predominantly from secondary-prevention populations and a smaller subgroup of high-risk primary prevention patients [2,99]. In the Reduction of Cardiovascular Events with Icosapent Ethyl–Intervention Trial (REDUCE-IT), purified eicosapentaenoic acid (EPA) reduced MACE by approximately 25% among high-risk patients with controlled LDL-C and elevated triglycerides [99]. The cardiovascular benefit observed with IPE in REDUCE-IT was not fully explained by triglyceride lowering alone, although the contribution of purified EPA-specific effects, achieved EPA exposure, and the mineral-oil comparator remains debated, particularly in light of the neutral STRENGTH trial [2,100]. Accordingly, IPE may be considered in appropriately selected statin-treated patients with persistent hypertriglyceridemia who resemble the REDUCE-IT population, which included predominantly patients with established ASCVD and a smaller primary prevention cohort with diabetes and additional cardiovascular risk factors [2,99,101].

In contrast, fibrates have not consistently reduced cardiovascular events when added to statin therapy. PROMINENT demonstrated that substantial reductions in triglycerides and remnant cholesterol did not translate into cardiovascular benefit when apoB-containing particle burden was not reduced [24]. These findings suggest that improvement in triglyceride- or remnant cholesterol-associated biomarkers alone is insufficient, and that effective treatment of residual lipid risk may require a net reduction in apoB-containing lipoproteins [18,24].

Lipoprotein apheresis may also be considered in selected patients with severe hypercholesterolemia, particularly familial hypercholesterolemia, who remain substantially above LDL-C targets despite maximally tolerated lipid-lowering therapy [2]. This extracorporeal approach produces marked acute reductions in circulating apoB-containing lipoproteins and can serve as an adjunctive option for patients with severe or refractory lipid disorders when pharmacological therapy is insufficient [102].

### 6.2. Targeting Residual Inflammatory Risk

Persistent elevation of hsCRP despite optimal lipid-lowering therapy identifies a residual inflammatory phenotype, particularly among patients with established ASCVD, although its role as a prospective treatment-selection biomarker remains incompletely established [53,103]. CANTOS provided proof of concept that IL-1β inhibition, with downstream reductions in IL-6 and CRP, can reduce recurrent cardiovascular events independently of LDL-C lowering [14]. The association between on-treatment hsCRP reduction and clinical benefit is discussed in Section 5.4 [86].

Low-dose colchicine is U.S. Food and Drug Administration (FDA)-approved to reduce the risk of myocardial infarction, stroke, coronary revascularization, and cardiovascular death in adults with established atherosclerotic disease or multiple cardiovascular risk factors [104]. The Colchicine Cardiovascular Outcomes Trial (COLCOT) and the Low-Dose Colchicine 2 (LoDoCo2) trial demonstrated reductions in recurrent cardiovascular events among patients with recent myocardial infarction and chronic coronary disease, respectively [45,105]. The 2023 AHA/ACC Guideline for the Management of Patients with Chronic Coronary Disease supports consideration of low-dose colchicine as an adjunct to guideline-directed medical therapy in appropriately selected patients with chronic coronary disease [106]. However, benefit has not been consistent across clinical settings; CLEAR-SYNERGY did not demonstrate a significant reduction in its primary cardiovascular endpoint when colchicine was initiated soon after acute myocardial infarction [107].

Differences across anti-inflammatory trials suggest that cardiovascular benefit may depend on both the inflammatory pathway targeted and the population studied [14,46]. In the Cardiovascular Inflammation Reduction Trial (CIRT), low-dose methotrexate did not reduce IL-1β, IL-6, or hsCRP concentrations and did not reduce cardiovascular events among patients with stable atherosclerosis and diabetes or metabolic syndrome [46]. Unlike CANTOS, CIRT did not require elevated hsCRP for enrollment, suggesting that both pathway-specific inhibition and identification of patients with residual inflammatory risk may influence therapeutic response [14,46].

Direct inhibition of IL-6 remains a biologically compelling strategy for residual inflammatory risk, although cardiovascular outcomes benefit has not been established [108,109]. Ziltivekimab produced marked reductions in hsCRP, fibrinogen, and other inflammatory biomarkers in earlier studies, demonstrating substantial target engagement [108,110]. However, preliminary topline results from the phase 3 ZEUS trial, reported by the sponsor, indicated that ziltivekimab did not significantly reduce MACE in patients with established ASCVD, chronic kidney disease, and systemic inflammation [109]. Additional phase 3 trials are evaluating ziltivekimab in other cardiovascular populations [109,111]. These findings illustrate that favorable biomarker effects and biological target engagement do not necessarily translate into cardiovascular outcomes benefit and confirmatory outcomes trial data are required [108,109].

### 6.3. Targeting Lipoprotein(a)-Mediated Risk

Management of elevated Lp(a) currently focuses on intensive LDL-C lowering and comprehensive control of other modifiable cardiovascular risk factors in both primary- and secondary-prevention settings, with treatment intensity guided by overall cardiovascular risk until dedicated Lp(a)-lowering therapies demonstrate cardiovascular benefit [2]. Lipoprotein apheresis may provide an additional therapeutic option for selected patients with markedly elevated Lp(a) and progressive ASCVD despite comprehensive risk-factor management [52,102]. Apheresis acutely lowers both Lp(a) and LDL-C, although its invasive nature, need for repeated treatment, and limited availability restrict its use to highly selected patients [102]. Several RNA-targeted therapies have produced profound and sustained reductions in circulating Lp(a) [52,75]. Pelacarsen and siRNAs, including olpasiran, lepodisiran, and zerlasiran, have demonstrated substantial dose-dependent reductions in Lp(a), reaching approximately 80% with pelacarsen and exceeding 80% with several siRNA regimens [75,79,80,81]. Muvalaplin, an oral small-molecule inhibitor of Lp(a) assembly, offers a potentially convenient noninjectable approach [112].

Large cardiovascular outcomes trials, including Lp(a)HORIZON, OCEAN(a), and ACCLAIM, are evaluating whether selective Lp(a) lowering reduces cardiovascular events [76,77,78]. Until these trials report, the substantial reductions achieved with emerging therapies should be regarded as favorable biomarker changes rather than evidence of clinical benefit [75,76,77,78,79,80,81,113]. Positive results would establish Lp(a) not only as a causal risk factor and measurable biomarker but also as an outcomes-validated therapeutic target [52,76,77,78].

## 7. Clinical Application of Biomarker-Informed Risk Assessment and Management

This section summarizes practical considerations for biomarker assessment, interpretation, and therapeutic decision-making based on current evidence and contemporary guideline recommendations (Table 2).

### 7.1. A Biomarker-Informed Approach to Residual Cardiovascular Risk

In patients with persistent cardiovascular risk despite achievement of LDL-C goals, complementary biomarkers should be selected according to the clinical context and the biological pathways that may be contributing to residual risk [2,54]. ApoB and remnant cholesterol may help identify persistent atherogenic lipoprotein burden, whereas Lp(a) and hsCRP may characterize genetically mediated and inflammatory residual-risk phenotypes, respectively [18,27,52,53]. Current guidelines consider apoB measurement reasonable in adults receiving lipid-lowering therapy, particularly those with ASCVD, cardiovascular-kidney-metabolic syndrome, type 2 diabetes, and/or elevated triglycerides, to guide further therapeutic intensification once LDL-C and/or non-HDL-C goals have been achieved [2]. Lp(a) should generally be measured at least once during adulthood because its concentration is predominantly genetically determined and relatively stable over time [2,11,52]. In contrast, hsCRP may be measured in clinically stable patients to identify residual inflammatory risk, with repeat testing when markedly elevated values suggest an acute inflammatory process [53,56].

### 7.2. Integrating Biomarkers into Therapeutic Decision-Making

Therapeutic decisions should connect biomarker-defined phenotypes to interventions supported by the corresponding level of evidence (Section 6; Table 2) [54]. Persistent elevation of apoB despite achievement of LDL-C and/or non-HDL-C goals may support consideration of further intensification of established lipid-lowering therapy in appropriately selected patients [2]. Persistent hypertriglyceridemia or elevated remnant cholesterol may help identify residual lipid risk and should prompt assessment of the underlying metabolic phenotype; IPE may be considered in eligible patients with persistent hypertriglyceridemia who resemble those enrolled in REDUCE-IT [18,99,100]. In selected patients with chronic coronary disease, low-dose colchicine may be considered as an adjunct to guideline-directed medical therapy for secondary prevention of recurrent ASCVD events [106]. IL-6-targeted therapies remain investigational because cardiovascular outcomes benefit has not been established [109]. Elevated Lp(a) should currently prompt comprehensive optimization of modifiable cardiovascular risk factors, with lipoprotein apheresis considered in highly selected patients with markedly elevated Lp(a) and progressive ASCVD [52,102]. Biomarkers without validated therapeutic implications should primarily refine risk assessment rather than directly determine treatment [54]. Because these phenotypes may coexist, therapeutic decision-making should integrate multiple biomarkers with the overall clinical context rather than rely on a single laboratory value [54].

### 7.3. Biomarker-Informed Clinical Framework

**Figure 2:**
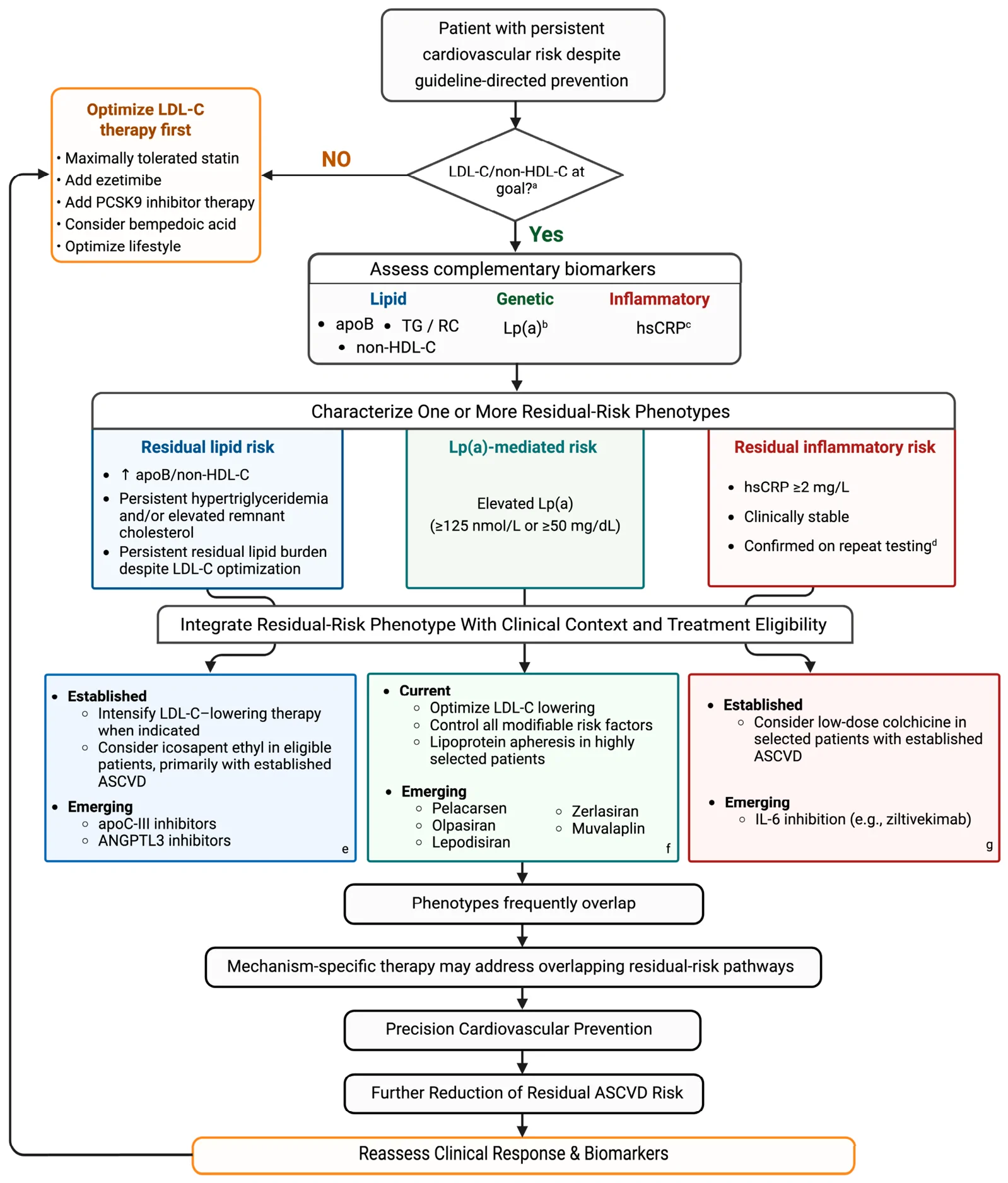
Biomarker-informed clinical algorithm for residual cardiovascular risk assessment and management. a, Per the 2026 ACC/AHA Guideline recommendations. b, Lp(a) is generally measured once during adulthood. c, Interpret hsCRP only in clinically stable individuals. d, Repeat hsCRP testing to confirm persistent elevation when appropriate. e, Evidence: REDUCE-IT. f, Trials: Lp(a)HORIZON, OCEAN(a), ACCLAIM-Lp(a). g, Evidence: COLCOT, LoDoCo2. Evidence for mechanism-specific therapies is strongest in secondary-prevention populations with established ASCVD; primary prevention evidence is more limited. **Abbreviations:** ACC/AHA, American College of Cardiology/American Heart Association; LDL-C, low-density lipoprotein cholesterol; non-HDL-C, non-high-density lipoprotein cholesterol; PCSK9, proprotein convertase subtilisin/kexin type 9; apoB, apolipoprotein B; TG, triglycerides; RC, remnant cholesterol; Lp(a), lipoprotein(a); hsCRP, high-sensitivity C-reactive protein; ASCVD, atherosclerotic cardiovascular disease; apoC-III, apolipoprotein C-III; ANGPTL3, angiopoietin-like protein 3; IL-6, interleukin-6.

Figure 2 presents a practical framework for biomarker-informed assessment and management of residual cardiovascular risk, integrating biomarker interpretation with therapies according to the strength of evidence supporting cardiovascular benefit.

### 7.4. Practical Considerations and Current Limitations

Several practical limitations should be considered before widespread implementation of biomarker-informed management [54]. ApoB measurement is standardized and widely available for clinical use [2]. In contrast, Lp(a) measurement remains subject to assay-related challenges related to apo(a) isoform size and differences in reporting units [11,52]. Although hsCRP is inexpensive and broadly accessible, it should be interpreted only in clinically stable patients because acute inflammatory conditions may substantially increase its concentration [53,56].

Prospective randomized evidence comparing a comprehensive biomarker-informed strategy with conventional LDL-C-guided care remains lacking [54]. Ongoing clinical and cardiovascular outcomes studies will further clarify whether interventions targeting triglyceride-rich lipoprotein metabolism, IL-6 signaling, or Lp(a) translate favorable biomarker changes into clinically meaningful cardiovascular benefit [74,76,77,78,109].

## 8. Future Directions

Despite substantial advances in understanding residual cardiovascular risk, several translational priorities will determine whether emerging biological insights can be incorporated into clinical practice [54,115].

### 8.1. Emerging Mechanism-Specific Therapies

Emerging therapies increasingly target specific biological drivers of residual cardiovascular risk [10,53]. Therapies targeting apoC-III and ANGPTL3 have shown promising effects on triglyceride-rich and apoB-containing lipoproteins, although cardiovascular outcomes benefit remains unestablished [18,114]. ApoC-III inhibition has advanced clinically, with olezarsen approved as an adjunct to diet to reduce triglycerides in adults with familial chylomicronemia syndrome and to reduce triglycerides and the risk of acute pancreatitis in adults with severe hypertriglyceridemia; however, these indications should be distinguished from ASCVD risk reduction because cardiovascular outcomes benefit has not been demonstrated [116]. Similarly, IL-6 inhibition and other inflammation-directed strategies remain investigational, with recent neutral cardiovascular results from ZEUS highlighting the need for outcomes validation despite substantial biomarker improvement [109,111]. For Lp(a)-mediated risk, ongoing phase 3 trials will determine whether selective Lp(a) lowering becomes an outcomes-validated therapeutic strategy, while oral agents such as muvalaplin may expand future treatment options [76,77,78,112]. Collectively, the clinical role of these therapies will depend on randomized evidence demonstrating cardiovascular benefit beyond favorable biomarker changes [54,109].

### 8.2. Gene Editing and Durable Genomic Therapies

Gene-editing technologies offer the possibility of durable treatment for genetically mediated lipid disorders [117,118]. In a first-in-human phase 1 study, a single intravenous dose of VERVE-102, an in vivo adenine base-editing therapy targeting hepatic PCSK9, produced dose-dependent and durable reductions in circulating PCSK9 and LDL-C [119]. Additional approaches targeting lipid-related pathways, including ANGPTL3, remain under development [120]. However, off-target effects, delivery specificity, immunogenicity, potentially permanent genomic changes, long-term safety, and ethical and regulatory considerations remain important barriers [120]. These approaches remain investigational and will require larger studies with longer follow-up to establish long-term safety, durability, and clinical benefit [119,120]. Although current gene-editing strategies have focused largely on lipid lowering, they illustrate the broader potential of durable, mechanism-specific therapies in future precision cardiovascular prevention [120].

### 8.3. Multi-Omic Precision Cardiovascular Prevention

As discussed in Section 5.5, emerging biomarkers such as GlycA, proteomic and metabolomic profiling, and PRS have shown promise for improving cardiovascular risk stratification [55,60,87,88]. Looking ahead, precision cardiovascular prevention may increasingly integrate these complementary approaches with conventional biomarkers and clinical characteristics to further individualize cardiovascular risk assessment [54,55,96,115].

### 8.4. Biomarker-Guided Therapeutic Trials and Remaining Knowledge Gaps

A major research priority is to determine whether a comprehensive strategy that integrates apoB, remnant cholesterol, hsCRP, and Lp(a) with evidence-based, pathway-specific therapeutic decisions improves cardiovascular outcomes compared with conventional LDL-C-guided care [54,115]. Future trials should determine which biomarkers most effectively identify patients likely to benefit from mechanism-specific therapies and establish appropriate biomarker thresholds, treatment targets, testing intervals, and implementation strategies [54]. These studies should also evaluate cost-effectiveness, treatment burden, adherence, patient preferences, and health equity [54,115]. Ultimately, biomarker-guided precision prevention will require evidence that it improves not only risk classification but also therapeutic decision-making and cardiovascular outcomes across diverse populations [54].

## 9. Conclusions

Despite the success of intensive LDL-C lowering, substantial cardiovascular risk persists through heterogeneous lipid-mediated, genetically determined, and inflammatory pathways [9,17]. Complementary biomarkers such as apoB, remnant cholesterol, Lp(a), and hsCRP may help characterize these sources of residual risk, but their clinical significance should be interpreted according to distinct levels of evidence for prognostic value, causal relevance, and therapeutic actionability [18,27,52,53,54]. Current management should prioritize interventions with demonstrated cardiovascular outcomes benefit while avoiding the assumption that favorable biomarker changes necessarily translate into fewer cardiovascular events [54,109]. Ultimately, the future of cardiovascular prevention will require not only achieving progressively lower LDL-C levels but also determining whether identification and treatment of the biological pathways underlying residual risk translate into improved cardiovascular outcomes [2,17,54].

## Figures and Tables

**Table 1:** Clinically relevant biomarkers for assessment of residual cardiovascular risk after achievement of LDL-C goals. Guideline status and clinical utility are summarized from contemporary guidelines, consensus statements, and biomarker reviews [2,11,18,27,50,51,52,53,54,55,56,57,58,59,60,61,62,63,64,65].

Biomarker	What It Reflects	When Is It Most Useful?	Current Guideline Status
**Clinically Established Biomarkers for Risk Assessment**			
LDL-C	LDL cholesterol burden	Routine assessment in all patients	Primary therapeutic target
Non-HDL-C	Total cholesterol in apoB-containing lipoproteins	Hypertriglyceridemia, diabetes, metabolic syndrome	Guideline-recognized secondary target in selected patients
apoB	Circulating atherogenic particle number	ASCVD, CKM syndrome, type 2 diabetes, hypertriglyceridemia, or discordance between LDL-C and apoB-defined particle burden	Reasonable adjunct marker for risk assessment and treatment guidance in selected patients
Remnant Cholesterol (RC)	Cholesterol within triglyceride-rich remnant lipoproteins	Persistent hypertriglyceridemia, cardiometabolic disease, residual risk despite LDL-C control	Useful marker of residual lipid risk; not currently a formal therapeutic target
Lp(a)	Genetically determined lifelong atherothrombotic risk	Premature ASCVD; familial hypercholesterolemia; strong family history; recurrent events despite LDL-C lowering	Recommended at least once during adulthood
hsCRP	Residual vascular inflammation	Residual inflammatory risk assessment	Risk-enhancing factor when hsCRP ≥ 2 mg/L on repeated measurements
**Emerging Biomarkers**			
GlycA	Integrated inflammatory and metabolic signal	Investigational	Not recommended for routine clinical use
Proteomics	Multi-protein cardiovascular risk signatures	Investigational	Research use only
Metabolomics	Metabolic pathway profiling	Investigational	Research use only
Polygenic Risk Scores	Inherited genetic susceptibility	Younger individuals; strong family history	Not recommended for routine clinical use
NETosis-related biomarkers	NET formation and thromboinflammatory activity	Investigational	Not recommended for routine clinical use

**Abbreviations:** LDL-C, low-density lipoprotein cholesterol; non-HDL-C, non-high-density lipoprotein cholesterol; apoB, apolipoprotein B; CKM, cardiovascular-kidney-metabolic; RC, remnant cholesterol; Lp(a), lipoprotein(a); ASCVD, atherosclerotic cardiovascular disease; hsCRP, high-sensitivity C-reactive protein; PRS, polygenic risk scores; NET, neutrophil extracellular trap.

**Table 2:** Biomarker-informed therapeutic strategies for residual cardiovascular risk after achievement of LDL-C goals. Therapeutic evidence is strongest in secondary-prevention populations with established ASCVD; primary prevention evidence is more limited. Biomarkers characterize residual-risk phenotypes but should not be interpreted as stand-alone treatment-selection criteria. Evidence summarized from randomized cardiovascular outcomes trials, contemporary guidelines, and ongoing phase 3 studies [2,52,76,77,78,99,102,105,106,109,111,112,113,114].

Residual Risk Phenotype	Biomarker(s)	Current Management	Emerging Therapies	Evidence Status
Residual lipid risk	apoB, non-HDL-C, remnant cholesterol, triglycerides	Intensification of lipid-lowering therapy; icosapent ethyl in eligible patients, primarily those with established ASCVD	apoC-III inhibitors; ANGPTL3 inhibitors	REDUCE-IT demonstrated cardiovascular benefit; cardiovascular benefit of apoC-III and ANGPTL3 inhibition remains unestablished
Residual inflammatory risk	hsCRP	Consider low-dose colchicine in selected patients with chronic coronary disease; IL-6-targeted therapies remain investigational	IL-6-targeted therapies (e.g., ziltivekimab)	Colchicine benefit established; ZEUS neutral; other IL-6 trials ongoing
Lp(a)-mediated risk	Lp(a)	Intensive LDL-C lowering and comprehensive risk-factor management; consider lipoprotein apheresis in highly selected patients	Pelacarsen, olpasiran, lepodisiran, zerlasiran, muvalaplin	Marked Lp(a) reductions demonstrated; cardiovascular outcomes trials are ongoing

**Abbreviations:** apoB, apolipoprotein B; non-HDL-C, non-high-density lipoprotein cholesterol; ASCVD, atherosclerotic cardiovascular disease; apoC-III, apolipoprotein C-III; ANGPTL3, angiopoietin-like protein 3; hsCRP, high-sensitivity C-reactive protein; IL-6, interleukin-6; Lp(a), lipoprotein(a); LDL-C, low-density lipoprotein cholesterol.

## Data Availability

No new data were created or analyzed in this study. Data sharing is not applicable to this article.
